# Acoustic Imaging Method for Gas Leak Detection and Localization Using Virtual Ultrasonic Sensor Array

**DOI:** 10.3390/s24051366

**Published:** 2024-02-20

**Authors:** Mu Liang, Kuan Yang, Mingyang Feng, Kaijun Mu, Mingqi Jiao, Lei Li

**Affiliations:** School of Physics and Microelectronics, Zhengzhou University, Zhengzhou 450001, China

**Keywords:** virtual ultrasonic sensor array, gas leakage, virtual beamforming, imaging detection and localization

## Abstract

An acoustic imaging method for detecting and locating gas leaks based on a virtual ultrasonic sensor array is proposed and experimentally demonstrated. A scanning sensor array of only two sensors is used to collect the acoustic signals generated by the leakage hole. The matrix of the leakage signal is processed by the cross-power spectrum method to achieve time consistency, afterward, the location of the leakage source can be calculated by the virtual beamforming method. The influence of the number of sensors and the distance between adjacent sensors on the effect of the proposed method are compared and discussed. To verify the effectiveness and operability of the detection and localization method, several experiments were carried out. Furthermore, a series of experiments were conducted to assess the accuracy and stability of this method. The experimental results demonstrate that the proposed method based on a virtual sensor array can achieve highly accurate localization of gas leaks and performs well regarding stability.

## 1. Introduction

The transportation and storage of gases, which include natural gas, carbon dioxide, and so on, are indispensable in industrial applications and people’s daily lives [1,2,3,4]. Gas leaks generated in transport or storage tools, such as gas pipes and pressure vessels, can cause many serious problems because of several factors, including corrosion, aging, and third-party damage [5,6]. Therefore, quickly detecting gas leaks and accurately locating the gas leak source has become an urgent problem.

In the field of fault detection, imaging gas leakage detection methods have obvious advantages of high efficiency, dynamic intuition, and large information content [7,8]. Currently, various methods for detecting and locating gas leak imaging have been proposed, such as thermal imaging, backscatter imaging, and multi-spectral imaging [9,10]. Thermal imaging [11] detects small differences in temperature by using expensive thermal imagers. Another disadvantage of thermal imaging is that the accuracy will be seriously affected if the temperature of the escaping gas is close to the ambient temperature. For backscatter imaging [12], expensive equipment costs limit the application of this technology. For multi-spectral imaging [13], the disadvantages are that it is selective for gases and requires expensive imagers. Photoacoustic imaging based on photoacoustic spectroscopy [14,15,16] is sensitive enough and may provide a new way for gas leak detection. However, this method is only suitable when the type of leaking gas is already known because the gas absorption of the photoacoustic spectroscopy is specific. Based on the above methods, it can be found that there is a lack of an imaging leakage detection method with a wide application range, no selectivity, and low cost.

For the high sensitivity, high localization accuracy, low false alarm rate, short testing time, and great adaptability, gas leak detection technology [17] based on acoustic emission (AE) technology has become the research hotspot of the gas leak detection field. When gas leaks occur, because of the large pressure difference between the inside and outside, the leaking gas rushes out of the leak hole at a high velocity to form turbulence, which is generated by the Reynolds stress or the shear forces and causes the air to sound. The sound frequency generated by the leakage source is mainly distributed from 10 kHz to 100 kHz, and the most obvious energy difference between leak signals and the environmental noise is at 40 kHz. Therefore, the 40 kHz is the preferred frequency for gas leak detection. According to the way of dealing with leakage signals, there are two types of typical AE methods: a detection method with a time domain and a localization method with a spatial domain [18,19,20]. In the time domain methods, leakage signals are collected by the acoustic sensors installed at both ends of gas pipelines [21]. Then, it estimates the location of the leak source based on the time difference between leak signals propagating to the different sensors [22,23]. The advantages of these methods are the fact that the localization accuracy is high and the system structure is simple. However, their localization accuracy is susceptible to various factors, including gas flow rate, ambient temperature, and gas pressure [24].

As with the other typical AE method, the spatial domain methods use an ultrasonic transducer array with a certain distance from the leaking pipe to collect leakage signals, and then use array signal processing technology to detect and locate gas leakage [25,26]. Yongyan et al. [27] used a liner-type array composed of six AE sensors to collect leakage signals and located the leak by the method based on near-field beamforming and acoustic emission techniques. Zhangyu et al. [28] first proposed an improved beamforming algorithm to obtain the power spectrum of the leaked signals collected by the L-shaped sensor array of eight Nano-30 sensors, and it can be seen that the location of the leakage from the power spectrum. Based on previous research, in 2018, a novel method for locating multiple leakage sources using a linear sensor array of four elements based on wavelet packet analysis and MUSIC algorithm was proposed [29]. That same year, a detecting and locating gas leakage method based on data fusion of energy decay and time difference of arrival, using a rectangular array of four ultrasonic sensors was proposed [30]. Compared with the contact measurement methods, the non-contact measurement methods further improve the accuracy of leakage localization and are more widely used; combined with the recent robust beamformer algorithms [31,32,33], the localization accuracy can be further improved. However, the localization accuracy is susceptible to the spacing between adjacent array elements and the number of array elements.

As a method of great adaptability, no selectivity, and cost, there are few reports about imaging leakage detection based on the acoustic emission method. The reason for this situation is that the imaging leakage detection method requires a large aperture sensor array to ensure the imaging resolution, while the use of large aperture sensor arrays will impose many limitations. Firstly, the localization accuracy depends upon their respective spatial positions (the geometry of the array) and the number of array elements. Limited by the volume of the array elements, the two-dimensional leak imaging based on the sensor array is difficult to realize. To overcome this problem, In [34], the ultrasonic transducers are inserted into the waveguide, reducing the effective interelement spacing to λ/2 for grating lobe-free beamforming. Secondly, excessive sensors in the array directly lead to very high system cost and complexity [35].

To address the need for a more efficient means of locating gas leaks, an acoustic imaging method for detecting and locating gas leakage based on the virtual phased array is first presented in this paper. The virtual phase array was proposed for the radar and sonar applications [36,37] and has been widely used in the filed of medical tomography [38], wi-fi holography [39], and acoustic imaging [40]. However, the visual phased array has not been used for gas leakage detection.

**Figure 1:**
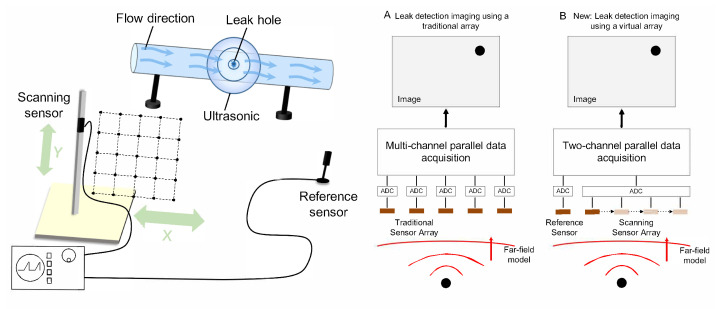
(**a**) Schematic diagram of experimental equipment. (**b**) Comparison of gas leak imaging methods based on traditional array and virtual array.

In this study, the virtual phased array technology is introduced into gas leak detection for imaging detection and localization. A scanning array consisting of two ultrasonic sensors is used to collect leak signals instead of a traditional array composed of multiple sensors. We sequentially scan with one sensor (scanning sensor) to record the amplitude data and the other sensor (reference sensor) is used to record the phase data. Then, the position of the leakage hole is estimated by combining the virtual beamforming and the cross-power spectrum methods. Our approach is presented in Figure 1a, and we can see the difference between our method and the traditional method in Figure 1b. As is shown in Figure 1b, the cost and complexity of the system based on this method have been greatly reduced because it reduces the number of sensors to only two and does not require a multi-channel synchronous data acquisition device. Furthermore, the array aperture can be larger than that of the conventional array to ensure the resolution of imaging detection and localization. In this study, the influence of the sensor number and the distance between adjacent sensors on the effect of the proposed method are compared and discussed. Several experiments were carried out in order to verify the effectiveness and operability of the localization method. Moreover, a series of experiments were conducted to evaluate the accuracy and stability of this method. The experimental results demonstrate that the detection and localization method based on the virtual sensor array can obtain highly accurate gas leak localization results and perform well regarding stability.

## 2. Method

### 2.1. Signal Model

With this scanning method, the signal $x_{i}\left(t\right)$ collected by the *i*-th element of the array can be given by

(1) $$x_{i}\left(t\right)=s\left(t-t_{pi}-\tau_{i}\right)\;\left(i=1,2,\cdots ,M\right).$$

Here, s(t) is the detected ultrasonic signal generated by the leak source. $t_{pi}$ denotes the time it takes for the leak signal to propagate from the leak source to each array element. $\tau_{i}$ is the scanning time interval between every element and the first element of the array. The number of array elements is described as *M*.

For the traditional method, the signal collected by the *i*-th element of the array can be given by

(2) $$x_{i}^{*}\left(t\right)=s\left(t-t_{pi}\right)\;\left(i=1,2,\cdots ,M\right).$$

A comparison between Equations (1) and (2) shows that the signals received by the scanning method have a scanning time interval $\tau_{i}$ compared with the signal received by the traditional method. The scanning time interval $\tau_{i}$ will interfere with the estimation of the location of the leakage hole. In order to estimate the location of the leak source, the scanning time interval $\tau_{i}$ needs to be eliminated first.

### 2.2. Signal Reconstruction

As shown in Figure 1a,b, a reference sensor is placed in a fixed position and works simultaneously with the scanning sensor to remove the interval time difference τ. The signal collected by the reference sensor can be written as follows:

(3) $$r_{i}\left(t\right)=s\left(t-t_{pr}-\tau_{i}\right)\;\left(i=1,2,\cdots ,M\right).$$

Here, $t_{pr}$ denotes the time it takes for the leak signal to propagate from the leak source to the reference sensor. The time difference between $r_{i}\left(t\right)$ and $x_{i}\left(t\right)$ is

(4) $$\Delta t_{i}=t_{pr}-t_{pi}.$$

According to Equation (4), the time difference between each element of the scanning array and the reference element does not include the time delay τ caused by the scanning interval. In our study, the cross-power spectrum method is used to remove scanning time interval τ and obtain the actual time difference between the two signals [41]. Firstly, perform Fourier transform on $x_{i}\left(t\right)$ and $r_{i}\left(t\right)$:

(5) $$\left\{\begin{array}{c} F_{x_{i}}\left(f\right)=\int_{-\infty }^{\infty }x_{i}\left(t\right)e^{-j2\pi ft}dt\\ \;\\ F_{r_{i}}\left(f\right)=\int_{-\infty }^{\infty }r_{i}\left(t\right)e^{-j2\pi ft}dt \end{array}\right.$$

Here:

(6) $$\int_{-\infty }^{\infty }x_{i}\left(t\right)e^{-j2\pi ft}dt=\int_{-\infty }^{\infty }r_{i}\left(t-\Delta t_{i}\right)e^{-j2\pi ft}dt=e^{-j2\pi f\Delta t_{i}}F_{r_{i}}\left(f\right)$$

The cross-power spectrum of $x_{i}\left(t\right)$ and $r_{i}\left(t\right)$ can be written as follows:

(7) $$Z\left(f\right)=F_{x_{i}}\left(f\right){F_{r_{i}}}^{*}\left(f\right)={\left|F_{r_{i}}\left(f\right)\right|}^{2}e^{j2\pi f\Delta t_{i}}$$

Here, ${F_{r_{i}}}^{*}\left(f\right)$ is a conjugate spectrum of $r_{i}\left(t\right)$. As is shown in Equation (7), Z(f) is only related to the signal frequency *f* and the time delay $\Delta t_{i}$ of the two signals. Therefore, the phase shift $\varphi_{i}$ between the signal of the *i*-th element and the signal of the reference element received simultaneously can be expressed as:

(8) φi=2πfΔti=arctanIm[Z(f)]Re[Z(f)]

According to Equation (4), $x_{i}\left(t\right)$ can be written as follows:

(9) $$x_{i}\left(t\right)=r_{i}\left(t\right)e^{-j\varphi_{i}}$$

Then $\varphi_{i}$ and the reference signal are used to reconstruct the array signal which textcolorreddoes not include τ. It can be seen from Figure 1a, that the two-dimensional rectangular virtual array of m×n elements is selected in our scheme. The signal received by the virtual array can be written as follows:

(10) $$X_{\left(m,n\right)}\left(t\right)=r_{\left(1,1\right)}\left(t\right)e^{-j\varphi_{\left(m,n\right)}}$$

where $\varphi_{\left(m,n\right)}$ is the phase difference between textcolorredthe reference signal and array signal calculated by the cross-power spectrum method. So all the signals received by the virtual array can be given by the following matrix form:

(11) $$X\left(t\right)=r_{\left(1,1\right)}\left(t\right)\times \left[\begin{array}{cccc} e^{-j\varphi_{\left(1,1\right)}} & e^{-j\varphi_{\left(1,2\right)}} & \cdots & e^{-j\varphi_{\left(1,n\right)}}\\ e^{-j\varphi_{\left(2,1\right)}} & e^{-j\varphi_{\left(2,2\right)}} & \cdots & e^{-j\varphi_{\left(2,n\right)}}\\ \vdots & \vdots & \ddots & \vdots \\ e^{-j\varphi_{\left(m,1\right)}} & e^{-j\varphi_{\left(m,1\right)}} & \cdots & e^{-j\varphi_{\left(m,n\right)}} \end{array}\right]$$

### 2.3. Imaging Localization Method

After eliminating the scanning time interval, the reconstructed signal and beamforming algorithm [42] can be used to estimate the location of the leak signal.

A narrowband beamformer output for each frequency bin of interest can be written as

(12) $$Y=W^{H}X$$

where ${\left(\cdot \right)}^{H}$ stands for conjugate transpose, Y denotes the beamformer output, and W is the beamformer weight vector.

Beam output power can be calculated as

(13) $$P=E\left[YY^{H}\right]=W^{H}RW$$

where E{·} is the statistical expectation, and R denotes the theoretical covariance matrix of the array output vector. R can be expressed as

(14) $$R=E[XX^{H}]$$

Let v denote the steering vector, and then we have

(15) $$v=\left[\begin{array}{cccc} e^{-jk^{T}p_{1,1}} & e^{-jk^{T}p_{1,2}} & \cdots & e^{-jk^{T}p_{1,n}}\\ e^{-jk^{T}p_{2,1}} & e^{-jk^{T}p_{2,2}} & \cdots & e^{-jk^{T}p_{2,n}}\\ \vdots & \vdots & \ddots & \vdots \\ e^{-jk^{T}p_{m,1}} & e^{-jk^{T}p_{m,2}} & \cdots & e^{-jk^{T}p_{m,n}} \end{array}\right]$$

where $p_{i,j}\left(i=1\cdots m,j=1\cdots n\right)$ stands for the spatial position of the array element and k is the wave number.

(16) $$k=\frac{\omega }{c}a=\frac{2\pi }{\lambda }a=\frac{2\pi }{\lambda }\left[\begin{array}{c} sin\theta cos\phi \\ sin\theta sin\phi \\ cos\theta \end{array}\right]$$

where a denotes the incident direction of the leak signal, ϕ is the pitch angle, and θ is the azimuth angle. Here ω is the signal angular frequency, and λ is the wavelength of the leakage signal.

System errors such as DOA mismatching, array element mutual coupling, signal source scattering, and so on will lead to mismatching of steering vectors in the actual experimental environment. The robust capon beamforming (RCB) has been employed to improve the robustness of adaptive beamforming with respect to any mismatches in steering vectors. We assume that the true steering vector is $v_{0}$ and the one being estimated is v, thus leading to the following constraint problem:

(17) $$\min_{v}v^{H}R^{-1}v\;s.t.\;{∥v-v_{0}∥}^{2}\leq \zeta$$

where ζ is the margin of error that is set by the user. Then, Equation (17) is transformed into an unconstrained problem by using the Lagrange multiplier method:

(18) $$F\left[v,\lambda \right]=v^{H}R^{-1}v+\lambda \left({∥v-v_{0}∥}^{2}-\zeta \right)$$

in which λ(λ≥0) is the Lagrange multiplier. Take the first derivative of Equation (18) with respect to v:

(19) $$R^{-1}v+\lambda \left(v-v_{0}\right)=0$$

By transforming Equation (19), the following can be obtained:

(20) $$v={\left(\frac{R^{-1}}{\lambda }+I\right)}^{-1}v_{0}$$

According to the matrix inversion theorem, Equation (20) can be further transformed into:

(21) $$v=v_{0}-{\left(I+\lambda R\right)}^{-1}v_{0}$$

Carry out the eigenvalue decomposition of R:

(22) $$R=U\Lambda U^{H}$$

where U is M×M matrix, and Λ stands for M×M diagonal matrix (M=m×n). By substituting Equation (22) into Equation (21):

(23) $$v=v_{0}-U{\left(I+\lambda \Lambda \right)}^{-1}U^{H}v_{0}$$

According to the obtained v, the weight vector of the RCB beamformer is:

(24) $$\begin{array}{cc} W_{RCB} & =\frac{R^{-1}v}{vR^{-1}v}\\ \; & =\frac{{\left(R+\frac{1}{\lambda }I\right)}^{-1}v_{0}}{v_{0}{\left(R+\lambda^{-1}I\right)}^{-1}R{\left(R+\lambda^{-1}I\right)}^{-1}v_{0}} \end{array}$$

The Lagrange multiplier λ can be obtained from Equation (25) by the Newton iteration method:

(25) $${∥v-v_{0}∥}^{2}={∥{\left(I+\lambda R\right)}^{-1}v_{0}∥}^{2}=\zeta$$

## 3. Numerical Simulation

To determine the optimal array configuration, according to the theory analysis of the imaging localization method, a numerical simulation based on MATLAB R2018a is carried out to discuss the influence of both the number of array elements and the distance of adjacent array elements on the localization of the algorithm.

### 3.1. Number of Array Elements

**Figure 2:**
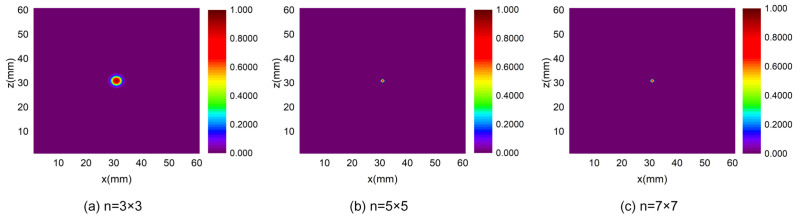
Energy distribution for the different number of array elements.

According to the imaging localization algorithm in the Method section, the MATLAB simulation for localization has been completed. We have carried out three sets of numerical experiments in which the number of array elements is set to 3×3, 5×5 and 7×7, respectively. The distance between adjacent array elements is 1mm and other parameters are controlled under the same conditions. Figure 2 is an energy distribution graph in which (a), (b), and (c) are the energy distribution map with 3×3, 5×5 and 7×7 array elements, respectively.

As can be seen from Figure 2, the performance of the localization algorithm becomes better as the number of array elements increases. In Figure 2a, the region gathering the highest energy (main lobe) and the maximum energy area is close to the leakage source; however, there are some other high-energy regions (side lobes) aside. These side lobes may affect the localization accuracy and lead to a wrong location. As is shown in Figure 2b,c, the more the number of array elements, the narrower the main lobe, and the smaller the number and energy amplitude of the highest energy gathering area. That is, the accuracy of localization has improved significantly as the number of array elements increases.

### 3.2. Distance between Adjacent Array Elements

To discuss the influence of the distance between adjacent array elements on the effect of the algorithm, we have carried out three sets of numerical experiments in which the distance between adjacent array elements is set to 1 mm, 2 mm, and 3 mm, respectively. The number of array elements is 3×3, and other parameters are the same.

**Figure 3:**
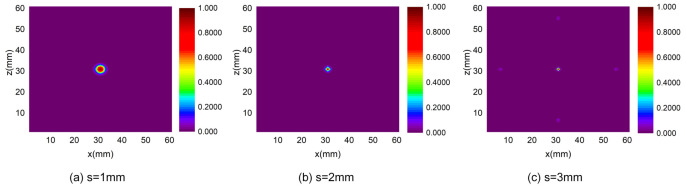
Energy distribution for different spacing between adjacent array elements.

Figure 3 is the energy distribution map. Although both Figure 3a,b show good performance in location, a pseudo leak source appears in Figure 3c. Comparing Figure 3a,b, it can be observed that the algorithm performance will deteriorate when the distance between adjacent array elements is narrower (Figure 3a). In Figure 3a, the main lobe interferes with the determination of the maximum energy area, thus increasing localization errors. On the other hand, there are two high-energy regions that appear in the energy distribution map when the distance between adjacent array elements is too large; the main lobe energy and side lobe energy are very close, which will interfere with the determination of the leak source (Figure 3c). When the distance between adjacent elements exceeds a half wavelength, the sidelobes will appear due to spatial aliasing. In order to avoid sidelobes and improve the localization resolution, the distance of half wavelength should be set as the spacing between adjacent array elements.

In conclusion, for the purpose of ensuring the accuracy of positioning, a large number of sensors are required. Furthermore, the spacing between adjacent array elements should be comprehensively considered to achieve the best imaging localization effect.

## 4. Experiments and Results

**Figure 4:**
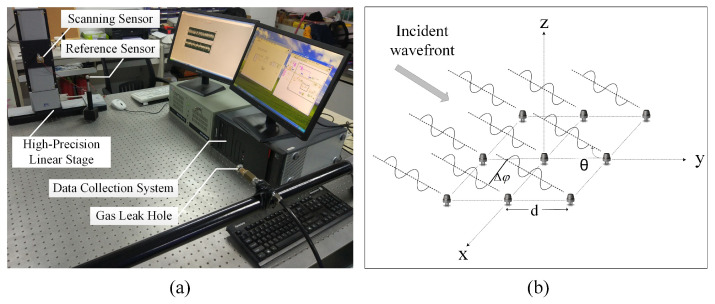
(**a**) Leakage detecting device. (**b**) The model of the far-field signal received by the array.

In order to verify the effectiveness and operability of the proposed method, we have carried out a series of experiments. The leakage detecting device is shown in Figure 4a. The nozzle in Figure 4a is a leakage source with the pressure difference between the inside and outside provided by an air compressor (QTS-1500X2, Outstanding Corporation, Taizhou, China). To simulate a leakage source, the air compressor causes the ejection of air from the nozzle, which is circular shaped with a 0.5 mm diameter. As can be seen from Figure 4a, there are two sensors for receiving leakage ultrasonic signals. One of the sensors (the scanning sensor) with a maximum scanning speed of 10 mm/s shifts in steps of 3 mm on a two-dimensional scanning translation stage (M-521.DG1, Physik Instrumente Corporation, Karlsruhe, Germany), whose scan area is 200 mm × 200 mm. The other sensor (the reference sensor) is in a fixed position. A sensor array of 20 × 20 elements is formed by the above device. The distance between the leak hole and the sensor array is 70 cm. A digital dual-channel recorder (PCI 8552, ART Technology Corporation, Beijing, China) is used to collect the signal data at the sampling rate of 1 MHz and then send the data to a PC. At last, MATLAB R2018a software is used to process the saved experimental data.

The frequency of leakage source signals is mainly distributed from 10 to 100 kHz, and the most obvious energy difference between the leakage signal and the environmental noise is at 40 kHz [43]. Thus, the signals with a frequency of 40 kHz are chosen to calculate the localization results.

To ensure the best response, the FUS-40CR piezoelectric ultrasonic sensors (Fuji Ceramics Corporation, Fujinomiya, Japan) are used to receive the leakage signals. The main technical specifications of the sensors are shown in Table 1.

### 4.1. Proof of Time-Stationary Sound Field

In this study, a time sharing scanning method was used to collect leakage signals, which is suitable for a time-stationary sound field. The location of the gas leak source is estimated by beamforming using the phase difference of the received signal. Here, the spatial characteristic of the received signal refers to the phase shift caused by the spatial variation. Therefore, before performing the localization calculation, the sound field needs to be proven to be time-stabilized, i.e., the phase shift in the signal is time-invariant.

Under the conditions of the time-stationary sound field, the phase differences are only related to the spatial position of different array elements, not to time. Assuming that the sound field is time-stable, the phase difference of the signals received by the two elements is constant and stable when the position of the two array elements is determined. Figure 4b is a schematic diagram of a two-dimensional rectangular sensor array receiving far-field signals and the signal propagation conforms to the plane wave model. As shown in Figure 4b, the distance between adjacent array elements is dsinθ, where θ is the incident direction of a plane wave and *d* is the distance between two adjacent elements. Theoretically, the phase shift between the received signals of adjacent arrays can be given by the following equation: Δφ=2πdsinθ/λ, where λ is the signal wavelength. The actual phase differences can be given by Equation (8).

**Figure 5:**
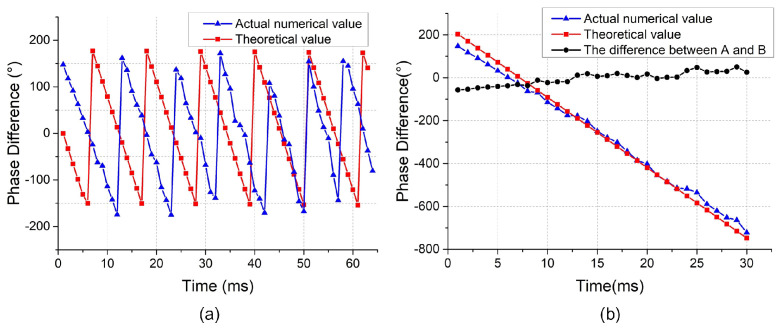
(**a**) The theoretical and actual values of the phase difference between the scanning sensor and the reference sensor. (**b**) Phase difference after unwrapping.

If the actual phase shift and the theoretical phase shift are the same, the leakage signals received can prove that they belong to a time-stationary sound field. The value of the actual phase difference and the theoretical phase difference are given in Figure 5. In Figure 5a, the actual numerical value is plotted by the blue line, and the theoretical value is plotted by the red line. The unwrapping phase shift curve is plotted in Figure 5b, and we can see that the slopes of the phase difference curve after unwrapping in the inset are −32.0 and −32.8, respectively. It can be seen from Figure 5 that there is the same change law between the value of actual and theoretical phase difference. Therefore, the leakage signals received can be proven to belong to a time-stationary sound field.

### 4.2. Results and Discussions

**Figure 6:**
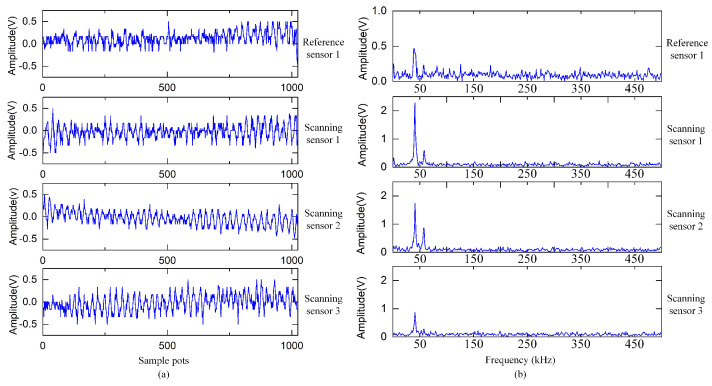
(**a**) Time domain signal waveforms. (**b**) Frequency spectra of the signals.

**Figure 7:**
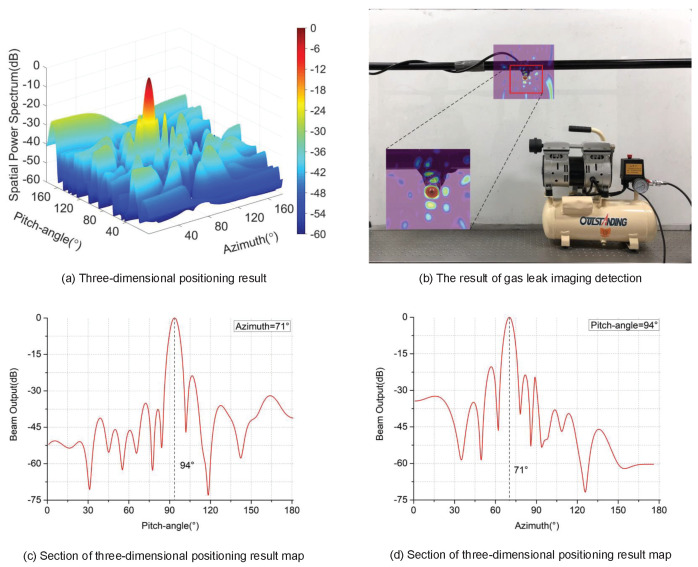
Gas leak localization result. (**a**) The spatial power spectrum of the leakage signal. (**b**) Location image of gas leakage source. (**c**,**d**) Section of the spatial power spectrum.

In this section, the effect of the proposed algorithm on the actual gas leak localization is studied. Figure 6a,b shows the signal waveforms in the time domain and frequency spectrum of the leakage signals received by the virtual sensor array, respectively. Figure 7 shows a typical localization result. Figure 7a illustrates the calculated spatial power spectrum, and the angle corresponding to the highest peak is the direction of the leak hole.

With the maximum output energy in the spatial power spectrum, the leak hole can be located at $\left(71^{\circ },\;94^{\circ }\right)$. As shown in Figure 7b, we fuse the two-dimensional display of the spatial power spectrum with the optical image of the experimental device to obtain the visual leakage detection results. According to Figure 7b, we can intuitively determine where the gas leakage occurs. Figure 7c,d are the profiles of the spatial power spectrum at the azimuth and pitch-angle, respectively.

Given the angle of the actual leak hole $\left(71^{\circ },\;94^{\circ }\right)$, these results demonstrate that the imaging detection method based on the virtual phased array is able to locate the leakage source effectively.

**Figure 8:**
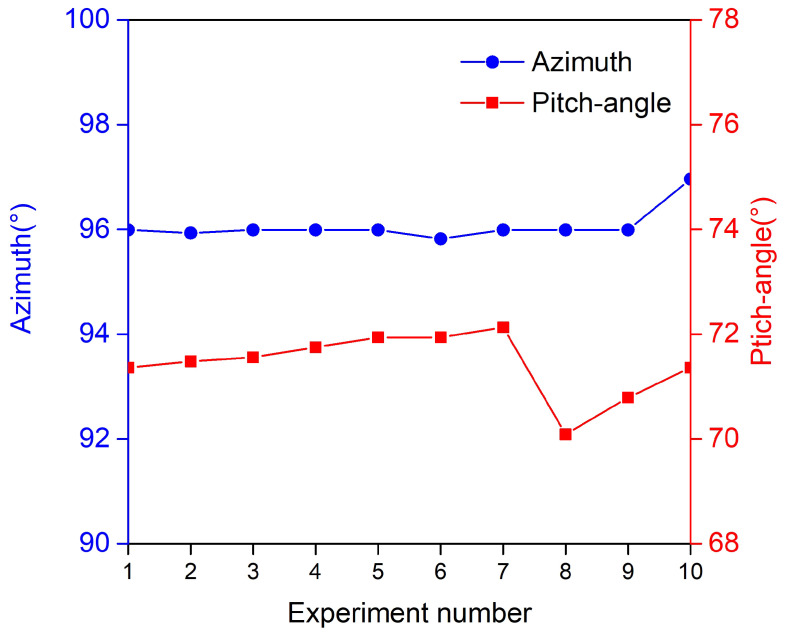
Localization error analysis.

In order to evaluate the accuracy and stability of the imaging detection and localization method based on the virtual phased array, the same experiments were repeated 10 times. The location errors are defined as the difference between the estimated leakage location and the actual leakage location listed in Table 2. Localization results are plotted in Figure 8. Given the location of the actual leak hole $\left(95^{\circ },\;71^{\circ }\right)$, as can be seen from Figure 8, all the measured results are around $\left(95^{\circ },\;71^{\circ }\right)$ and all the errors are around $\left(0.06^{\circ },\;0.43^{\circ }\right)$. The final measured locations of the leak hole by averaging the repeated experimental results are $\left(96.064^{\circ },\;71.44^{\circ }\right)$ with standard deviations of $\left(0.303^{\circ },\;0.578^{\circ }\right)$ and the averaging error is $\left(0.062^{\circ },\;0.428^{\circ }\right)$ with standard deviations of $\left(0.297^{\circ },\;0.610^{\circ }\right)$. These results suggest that the virtual sensor array method can obtain a high-accuracy gas leak localization result and perform well regarding stability.

## 5. Conclusions

A novel imaging method for gas leak detection and localization based on the virtual phased array is presented in this paper. The influences of various factors on the performance of the localization method are simulated, compared, and discussed, including the number of sensors and spacing between adjacent sensors in the array. The simulation results have suggested that the number of array elements should be large enough to maintain the localization accuracy, and the distance of half wavelength should be set as the spacing between adjacent array elements to avoid sidelobes and improve the localization resolution. Experiments are carried out to evaluate the effect of the virtual phased array technology on the actual gas leak, and the leak signals are collected by a scanning sensor array. The array signal is reconstructed by the cross-power spectrum, and the spatial power spectrum is obtained through the beamforming algorithm. Then, we fuse the two-dimensional display of the spatial power spectrum with the optical image of the experimental device to form a visual figure showing the leak source of the measured object. The results have demonstrated that a high resolution and visual leakage localization result can be given by this method. Furthermore, a series of experiments were conducted to verify the accuracy and stability of the method. The results are shown in Figure 8 and Table 2, which illustrates that the repeated experimental results are $\left(81.9^{\circ },\;20.40^{\circ }\right)$ with standard deviations of $\left(0.15^{\circ },\;0.18^{\circ }\right)$ and the averaging error is $\left(0.11^{\circ },\;0.39^{\circ }\right)$ with standard deviations of $\left(0.07^{\circ },\;0.05^{\circ }\right)$. The results demonstrate that the virtual sensor array method can obtain a high-accuracy gas leak localization result and performs well regarding stability.

## Figures and Tables

**Table 1:** Sensor main technical features.

Sensor Model	FUS-40CR
Diameter	15 mm
Directivity	50 deg
Sensitivity	−16 dB
Center frequency	40 kHz
Detection distance	0.2–6 m

**Table 2:** Localization result and error analysis.

Number	Result (°)		Error (°)	
	**Azimuth**	**Pitch-Angle**	**Azimuth**	**Pitch-Angle**
1	95.99	71.36	−0.01	0.36
2	95.93	71.36	−0.07	0.36
3	95.99	71.56	−0.01	0.56
4	95.99	71.15	−0.01	0.75
5	95.99	71.94	−0.01	0.94
6	95.82	71.94	−0.18	0.94
7	95.99	72.13	−0.01	1.13
8	95.99	70.09	−0.01	−0.91
9	95.99	70.79	−0.01	−0.21
10	95.96	71.36	−0.04	0.36
Mean (°)	96.064	71.44	0.062	0.428
Standard deviation (°)	0.303	0.578	0.297	0.610

## Data Availability

No new data were created or analyzed in this study. Data sharing is not applicable to this article.
